# Loss of secreted gelsolin enhances response to anticancer therapies

**DOI:** 10.1136/jitc-2022-005245

**Published:** 2022-09-26

**Authors:** Kok Haw Jonathan Lim, Evangelos Giampazolias, Oliver Schulz, Neil C Rogers, Anna Wilkins, Erik Sahai, Jessica Strid, Caetano Reis e Sousa

**Affiliations:** 1 Immunobiology Laboratory, The Francis Crick Institute, London, UK; 2 Department of Immunology and Inflammation, Imperial College London, London, UK; 3 Tumour Cell Biology Laboratory, The Francis Crick Institute, London, UK; 4 Division of Radiotherapy and Imaging, The Institute of Cancer Research, London, UK

**Keywords:** dendritic cells, antigen presentation, immunity

## Abstract

Type 1 conventional dendritic cells (cDC1) play a critical role in priming anticancer cytotoxic CD8^+^ T cells. DNGR-1 (a.k.a. CLEC9A) is a cDC1 receptor that binds to F-actin exposed on necrotic cancer and normal cells. DNGR-1 signaling enhances cross-presentation of dead-cell associated antigens, including tumor antigens. We have recently shown that secreted gelsolin (sGSN), a plasma protein, competes with DNGR-1 for binding to dead cell-exposed F-actin and dampens anticancer immunity. Here, we investigated the effects of loss of sGSN on various anticancer therapies that are thought to induce cell death and provoke an immune response to cancer. We compared WT (wildtype) with *Rag1^–/–^*, *Batf3^–/–^*, *Clec9a^gfp/gfp^*, *sGsn^–/–^* or *sGsn^–/–^ Clec9a^gfp/gfp^* mice implanted with transplantable tumor cell lines, including MCA-205 fibrosarcoma, 5555 Braf^V600E^ melanoma and B16-F10 LifeAct (LA)-ovalbumin (OVA)-mCherry melanoma. Tumor-bearing mice were treated with (1) doxorubicin (intratumoral) chemotherapy for MCA-205, (2) BRAF-inhibitor PLX4720 (oral gavage) targeted therapy for 5555 Braf^V600E^, and (3) X-ray radiotherapy for B16 LA-OVA-mCherry. We confirmed that efficient tumor control following each therapy requires an immunocompetent host as efficacy was markedly reduced in *Rag1^–/–^* compared with WT mice. Notably, across all the therapeutic modalities, loss of sGSN significantly enhanced tumor control compared with treated WT controls. This was an on-target effect as mice deficient in both sGSN and DNGR-1 behaved no differently from WT mice following therapy. In sum, we find that mice deficient in *sGsn* display enhanced DNGR-1-dependent responsiveness to chemotherapy, targeted therapy and radiotherapy. Our findings are consistent with the notion some cancer therapies induce immunogenic cell death (ICD), which mobilizes anticancer T cells. Our results point to cDC1 and DNGR-1 as decoders of ICD and to sGSN as a negative regulator of such decoding, highlighting sGSN as a possible target in cancer treatment. Further prospective studies are warranted to identify patients who may benefit most from inhibition of sGSN function.

**What is already known on this topic**

- Type 1 conventional dendritic cells (cDC1) play a non-redundant role in anticancer immunity.
- DNGR-1 is a dedicated cDC1 receptor that facilitates cross-presentation of dead-cell associated antigens.
- Secreted gelsolin (sGSN) is a physiological barrier to DNGR-1-dependent cross-presentation and dampens anticancer immunity.
- Some cancer therapies may induce an antitumor immune response by triggering immunogenic cell death.

**What this study adds**

- Loss of sGSN enhances therapeutic response to several immunogenic anticancer therapies in preclinical models including chemotherapy, targeted therapy and radiotherapy.

**How this study might affect research, practice or policy**

- Therapeutic inhibition of sGSN function, to unleash DNGR-1-dependent cross-presentation, could be explored as a component of more effective treatment regimens.

## Background

Type 1 conventional dendritic cells (cDC1) perform non-redundant functions in anticancer immunity.^1 2^ Mice deficient in cDC1s (eg, *Batf3^–/–^*) display lower spontaneous rejection of immunogenic tumors and decreased responses to T cell based immunotherapies.^1^ In humans, a cDC1 gene signature positively correlates with improved overall survival (OS) in patients with cancer and with responses to checkpoint blockade immunotherapy.^1 2^ A key role of cDC1 is to prime tumor antigen-specific CD8^+^ T cells. It is thought that this partly involves internalization by cDC1 of dead cancer cell remnants and subsequent MHC class I (cross-)presentation to CD8^+^ T cells of antigens derived from the debris.

DNGR-1 (a.k.a. CLEC9A) is a C-type lectin receptor ubiquitously expressed on cDC1 that binds to F-actin exposed on dead cell corpses and signals to enhance cross-presentation of dead-cell associated antigens.^3–6^ We recently found that secreted gelsolin (sGSN), an extracellular protein found in the plasma of all vertebrates,^7 8^ forms a natural barrier to DNGR-1-mediated cross-presentation.^9^ sGSN promotes cancer immune escape by competing with DNGR-1 for binding to extracellular F-actin and inhibiting DNGR-1-dependent tumor control.^9^ In humans, high expression of *CLEC9A* (encoding DNGR-1) in the tumor microenvironment is associated with greater survival and correlates with gene signatures of immune-mediated cancer control.^10^ In mice, DNGR-1 can contribute to CD8^+^ T cell-dependent control of transplantable tumors in animals deficient in sGSN.^9^

For decades, chemotherapy, radiotherapy and, more recently, targeted therapy, have been the mainstay of treatment for most patients with cancer. There is an increasing evidence that these therapies work not only by directly killing cancer cells but also by inducing or boosting antitumor immune responses.^11–13^ We; therefore, hypothesized that therapy-induced cell death would increase the antigenic visibility of tumors, augmenting DNGR-1 triggering by F-actin in *sGsn^–/–^* mice and resulting in improved anticancer immunity. Here, we show that mice deficient in *sGsn* display enhanced DNGR-1-dependent responsiveness to chemotherapy, radiotherapy and targeted therapy of tumors, highlighting sGSN as an attractive target for combinatorial cancer treatments that engage immunity.

## Methods

### Mice

*Batf3^–/–^*, *Clec9a^gfp/gfp^*, *sGsn^–/–^*, *sGsn^–/–^ Clec9a^gfp/gfp^*, *Rag1^–/–^* and WT (wildtype) mice on a C57BL/6 background were bred and maintained in specific-pathogen free conditions in the Biological Research Facility (BRF) at The Francis Crick Institute. In all experiments, male and female mice were used. Experiments were initiated when mice were between 6 and 14 weeks. In all experiments comparing groups of mice of different genotypes, they were co-housed with sex-matched and age-matched WT controls for at least 3 weeks to eliminate any microbiota-dependent effects. Alternatively, in some experiments where WT littermate controls were used, this is indicated in the figure legends. All animal experiments were performed on prospective approval of a study plan by the BRF at The Francis Crick Institute and adhered to the UK Animals (Scientific Procedures).^7^

### Transplantable tumors

Transplantable tumor cell lines used include MCA-205 fibrosarcoma (kind gift from George Kassiotis), 5555 Braf^V600E^ melanoma,^14^ and B16-F10 LifeAct (LA)-ovalbumin (OVA)-mCherry.^9^ All cell culture procedures were performed in a sterile condition in a laminar flow hood. Cultured syngeneic tumor cells were dissociated with trypsin (0.25%) (Gibco) and washed for three times in PBS (Gibco). The final cell pellet was resuspended in PBS for the required concentration of cells accordingly and injected subcutaneously (s.c.) in a volume of 100 µl in the shaved right flank of each mouse. Mice were monitored for tumor growth at least two to three times per week. The values of the longest (*l*) and perpendicular shortest (*w*) tumor diameters were measured using digital Vernier calipers (Mitutoyo), and tumor volume calculated using the formula: 0.5 x (*l x w^2^*),^15^ expressed in the standard metric units mm^3^. Mice-bearing tumors were monitored until the predetermined humane endpoint (eg, mean tumor diameter reaching ≥15 mm, weight loss ≥15%, or tumor ulceration) or for at least 30 days following tumor transplantation.

### Chemotherapy model

For in vivo chemotherapy, mice were inoculated s.c. with 0.5×10^6^ cells/100 µlμ MCA-205 fibrosarcoma. Doxorubicin (Sigma-Aldrich) was administered at a dose of 2.5 mg/kg in a final volume of 50 µl sterile PBS intratumorally. Treatment was administered when tumors became palpable, usually on day 6 or 7, when the mean tumor diameter reached 6–8 mm.

### Radiotherapy model

For radiotherapy, mice were inoculated s.c. with 0.2×10^6^ cells/100 µl of B16-F10 LA-OVA-mCherry melanoma. On day 7, mice received a single fraction of 10 Gy X-ray irradiation delivered in an X-ray irradiator cabinet (Xstrahl RS320 Research System). The machine was calibrated immediately prior to every use, and radiation was delivered at a consistent dose rate of 1.5–1.6 Gy/min (200kV, 16mA) at a field source distance of 20 cm. Radiation was targeted to the right flank tumor, precisely positioned using laser beams, through a 15 mm aperture at the center of an overlying customized lead shield (2 mm thickness). The shielded portion of the body received a maximum of 0.3 Gy radiation whereas 10 Gy was delivered to target tumor area. Mice received reversible general anesthesia throughout the duration of the procedure using a ‘sleep mix’ of fentanyl 0.05 mg/kg, midazolam 5 mg/kg and medetomidine 0.5 mg/kg, and reversed with a ‘wake mix’ of naloxone 1.2 mg/kg, flumazenil 0.5 mg/kg and atipam 2.5 mg/kg, administered intraperitoneally and dosed according to weight per mouse. Mice were kept in a warming chamber (35°C–37°C) (Datesand Thermacage) during anesthesia, both in preparation for administration of radiation and also for recovery. Mice receiving sham-irradiation were put through reversible anesthesia and recovery in the warming chamber for the same duration.

### Targeted therapy model

Mice received 0.2×10^6^ cells/100 µl s.c. inoculation of 5555 Braf^V600E^ melanoma. When tumors were palpable, generally at day 6 after tumor cell inoculation, mice received Braf^V600E^ kinase inhibitor PLX4720 (MedKoo) suspension at a dose of 45 mg/kg in total volume of 200–250 µl by oral gavage (o.g.) daily for a total of 2 weeks, with 5% DMSO as vehicle control.

### Statistics

Statistical analyses were performed using GraphPad Prism V.9 for Mac OS (V.9.3.0, updated 2021; San Diego, California, USA). Data are shown as mean±SE of the mean (SEM) unless otherwise stated. In general, for all tumor growth profiles, two-way analysis of variance was used to compare the means of two or more groups obtained at every single timepoint, and this was adjusted for multiple comparisons with post-hoc Bonferroni correction. The statistical differences annotated on the plots represent the comparison at the final timepoints within the respective follow-up period of each experiment. In all instances, p values are two tailed and p≤0.05 is considered the threshold for statistical significance. Where indicated in figures, the following scheme is used to represent the levels of statistical significance: *p≤0.05, **p<0.01, ***p<0.001, ****p<0.0001; ns, not significant.

## Results

### Loss of sGSN enhances response to immunogenic chemotherapy

**Figure 1:**
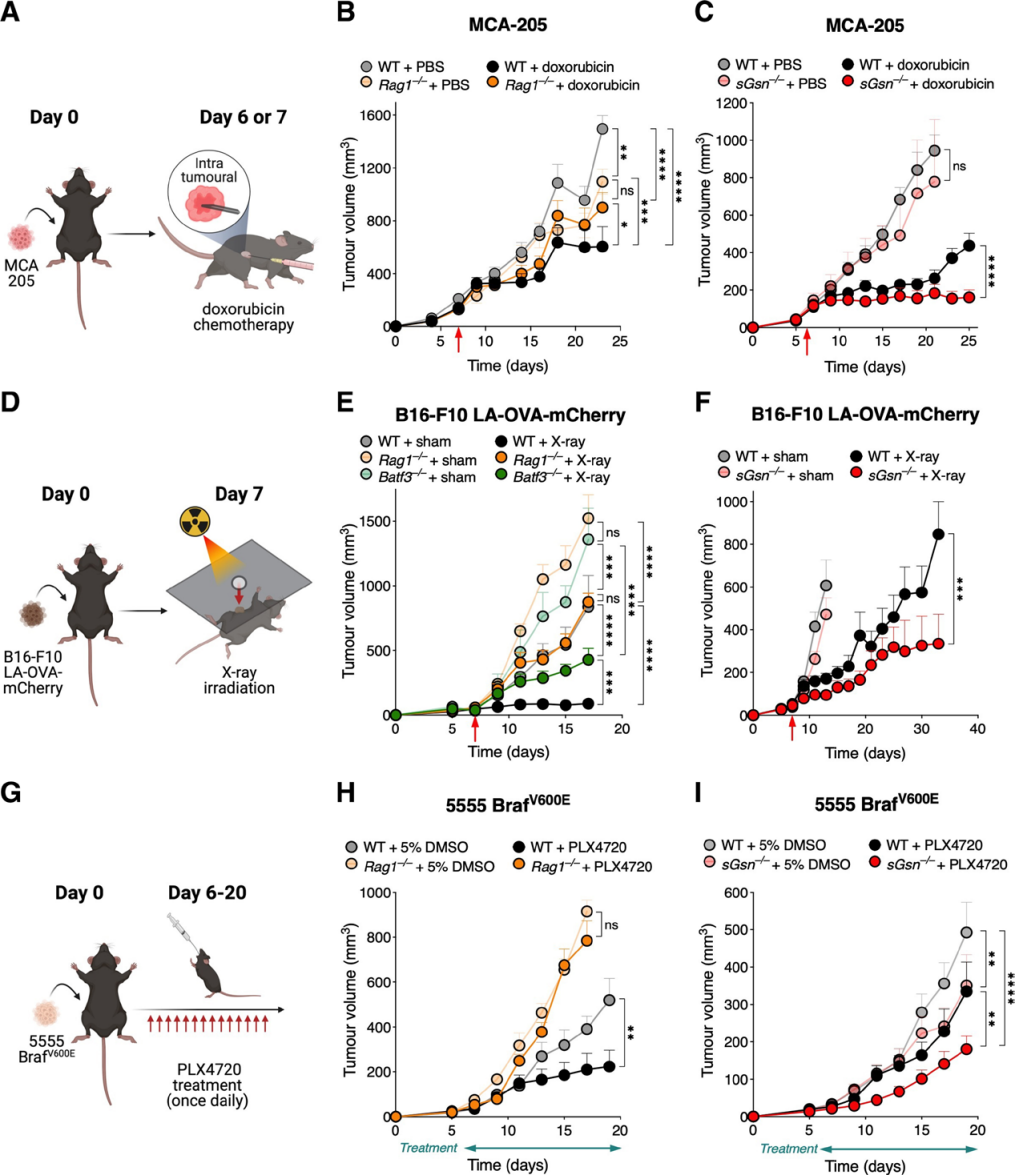
Loss of sGSN enhances response to immunogenic chemotherapy, radiotherapy, and targeted therapy. (A) Graphical illustration of the chemotherapy model for data shown in (B, C). Mice were subcutaneously inoculated with MCA-205 fibrosarcoma cells, and on day 6 or 7, randomized to receive either chemotherapy with doxorubicin or PBS vehicle control (as indicated by the red arrows in the plots). (D) Tumor growth profile in mice receiving either doxorubicin (WT, n=8; *Rag1^–/–^*, n=8), or vehicle control (WT, n=8; *Rag1^–/–^*, n=8). (C) Doxorubicin was administered to n=9 *sGsn^–/–^* mice vs n=11 WT littermates, and compared with n=4 *sGsn^–/–^* mice vs n=7 WT littermates receiving vehicle control. (D) Graphical illustration of the radiotherapy model for data shown in (E, F). Tumors were derived from subcutaneous inoculation of B16-F10 LA-OVA-mCherry melanoma cells into the shaved right flanks of mice. Mice were then randomized to receive a single fraction of x-ray irradiation to the target tumor area or sham irradiation (untreated), on day 7 following tumor implantation (as indicated by the red arrows in the plots). (E) Growth profile of tumors in WT (X-ray, n=10; sham, n=8), *Rag^–/–^* (X-ray, n=10; sham, n=10) and *Batf3^–/–^* (X-ray, n=10; sham, n=6) mice. (F) Growth profile of tumors in co-housed WT (X-ray, n=15; sham, n=12) and *sGsn^–/–^* (X-ray, n=14; sham, n=10) mice. (G) Graphical illustration of the targeted therapy model for data shown in (H, I). Mice were subcutaneously inoculated with 5555 BRAF^V600E^ melanoma cells, and on day 6, randomized to receive treatment with either the Braf-inhibitor PLX4720 or 5% DMSO as vehicle control, for a total of 14 days (as indicated by the treatment bars below the x-axes in the plots). (H) Growth profile of tumors in *Rag1^–/–^* mice (PLX4720, n=8; control, n=8) vs co-housed WT (PLX4720, n=7; control, n=6) mice. (I) Growth profile of tumors in co-housed WT (PLX4720, n=13; control, n=10) and *sGsn^–/–^* (PLX4720, n=14; control, n=12) mice. Data are represented as tumor volume (mm^3^) ±SEM, and groups were compared using two-way ANOVA with post hoc Bonferroni correction. where indicated, *p≤0.05, **p<0.01, ***p<0.001, ****p<0.0001; NS, not significant. Error bars are depicted in all plots; when not visible, errors are small. Data are representative of one experiment respectively for (C, F, H); one of two independent experiments for (B, E); and one of three independent experiments for (I). The data in (C, F) are further replicated in figure 2A, B, respectively. ANOVA, analysis of variance; LA, LifeAct; PBS, phosphate-buffered saline; OVA, ovalbumin; sGSN, secreted gelsolin; WT, wild type.

First, we confirmed that chemotherapy with doxorubicin in the transplantable MCA-205 primary fibrosarcoma requires an immunocompetent host for full effectiveness. Consistent with that notion, intratumoral doxorubicin administration reduced tumor growth in WT mice but therapeutic benefit was reduced in *Rag1^–/–^* mice which lack T and B cells (figures 1A, B). The efficacy of doxorubicin was similar in DNGR-1-deficient and WT mice, suggesting that DNGR-1 is dispensable in a sGSN-sufficient setting (online supplemental figure S1a). However, when tested in mice lacking sGSN, we observed that the administration of doxorubicin resulted in significantly greater tumor control compared with treated WT mice (figure 1A, C).

10.1136/jitc-2022-005245.supp1 Supplementary data

10.1136/jitc-2022-005245.supp2 Supplementary data

**Figure 2:**
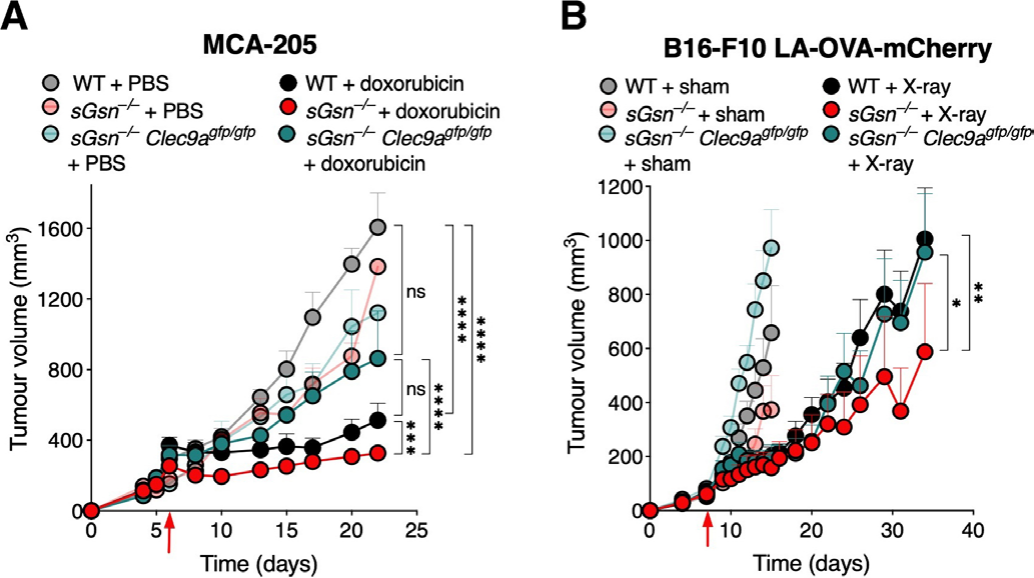
Enhanced therapeutic response in the loss of secreted gelsolin settings is dependent on DNGR-1. (A) Tumor growth profile in mice bearing MCA-205 fibrosarcoma receiving chemotherapy. Doxorubicin was administered to cohoused WT (n=10), *sGsn^–/–^* (n=10) and *sGsn^–/–^ Clec9a^gfp/gfp^* (n=10) mice, and compared with cohoused WT (n=10), *sGsn^–/–^* (n=3) and *sGsn^–/–^ Clec9a^gfp/gfp^* (n=3) mice receiving vehicle control. (B) Growth profile of B16-F10 LA-OVA-mCherry melanoma in co-housed WT (X-ray, n=14; sham, n=10), *sGsn^–/–^* (X-ray, n=11; sham, n=10) and *sGsn^–/–^ Clec9a^gfp/gfp^* (X-ray, n=12; sham, n=11) mice. Data are plotted as tumor volume (mm^3^) ±SEM, and mean tumor volumes were compared using two-way ANOVA with post hoc Bonferroni correction. Where indicated, *p≤0.05, **p<0.01, ***p<0.001, ****p<0.0001; NS, not significant. Error bars are depicted in all plots; when not visible, errors are small. Data are representative of one experiment respectively. ANOVA, analysis of variance; LA, LifeAct; OVA, ovalbumin; WT, wild type.

### The efficacy of immunogenic radiotherapy is improved in the absence of sGSN

We next tested the effect of sGSN loss in tumor responses to radiotherapy (figure 1D). We used the poorly immunogenic B16-F10 melanoma cell line engineered to express the model antigen OVA fused to mCherry and a 17-amino acid LA F-actin binding peptide (B16-F10 LA-OVA-mCherry).^9^ We found that effective and sustained control of B16-F10 LA-OVA-mCherry tumors following a single fraction of X-ray irradiation was lost in *Batf3^–/–^* mice lacking cDC1 or *Rag1^–/–^* mice lacking T and B cells (figure 1E). As in the doxorubicin chemotherapy model, tumor control following radiotherapy was unaffected by deficiency in DNGR-1 (online supplemental figure S1b). However, it was significantly enhanced in sGSN-deficient hosts (figure 1F).

### Targeted therapy induces immunogenic cell death and its efficacy is further enhanced in absence of sGSN

We also sought to investigate whether sGSN deficiency might benefit the response to targeted therapy with BRAF inhibitor in a mouse mutant-Braf melanoma model. Tumor-bearing mice implanted with 5555 Braf^V600E^ primary melanoma tumor cells were treated or not with PLX4720 (figure 1H). We found that the therapeutic effect of the drug was decreased in mice lacking T and B cells (figure 1H). Notably, 5555 Braf^V600E^ tumors were controlled to a greater extent in *sGsn^–/–^* compared with WT mice even in the absence of treatment with PLX4720 (figure 1I). Importantly, however, tumor control by PLX4720 was further enhanced by sGSN-deficiency (figure 1 and online supplemental figure S1c), confirming the importance of sGSN as a barrier to dead cell-induced immunity.

### The enhanced therapeutic response in *sGsn^–/–^* mice is DNGR-1-dependent

The data so far suggest that maximal therapeutic efficacy of some forms of chemotherapy, radiotherapy and targeted therapy in vivo requires an intact adaptive immune system and is further augmented by loss of sGSN. In order to validate that enhanced tumor control in *sGsn^–/–^* mice is DNGR-1-dependent, we tested some of the therapy models in mice deficient in both sGSN and DNGR-1. For both chemotherapy with intratumoral doxorubicin in MCA-205 and radiotherapy in B16-F10 LA-OVA-mCherry, mice deficient in both sGSN and DNGR-1 exhibited similar rate of tumor control to the respective treated WT controls, in contrast to the enhanced therapeutic responses observed in the treated *sGsn^–/–^* subgroups (figure 2A, B). Thus, sGSN impairs DNGR-1-dependent anticancer immunity-induced by therapy.

## Discussion

While much of the therapeutic effect of conventional anticancer therapies results from direct cytotoxicity to cancer cells, it is becoming clear that it can also include immune-dependent mechanisms. Specifically, it has been proposed that some cancer therapeutics can cause immunogenic cell death (ICD), allowing for sampling of tumor antigens by DCs that then prime or boost an antitumor T cell response.^11 12^ DNGR-1 is a receptor that marks the cDC1 subtype of DCs and plays a key role in coupling dead cell recognition to antitumor CD8^+^ T cell immunity.^3–6 16^ DNGR-1 activity is often masked by circulating sGSN such that the role of the receptor can be revealed only on loss of the latter protein.^9^ Here, we show that the immune-dependent efficacy of seemingly disparate therapies in a preclinical mouse model is increased by loss of sGSN. Notably, although DNGR-1 deficiency per se does not impact therapy efficacy, it curtails the therapeutic advantage conferred by loss of sGSN. Thus, these data implicate the sGSN-DNGR-1 axis in decoding ICD induced by cancer treatment.

The contribution of immunity to the effects of conventional cancer chemotherapy and radiotherapy is beginning to be appreciated in patients,^17^ but mechanistic insights have been primarily obtained in animal models. In mice, several chemotherapeutic agents, including the anthracycline doxorubicin, result in ICD and lead to antitumor responses that depend on DCs and CD8^+^ T cells.^12 18^ Similarly, both DCs and CD8^+^ T cells have been previously demonstrated to contribute to tumor control following radiotherapy.^13 19^ Our study provides further evidence for the immunogenicity of some anticancer therapies,^11 12^ showing that their potency is reduced in mice lacking T (and B) cells. We show that this immune benefit can be further enhanced in a DNGR-1 dependent manner on loss of sGSN, concordant with our previous work showing and increase in tumor-specific T cell responses in GSN-deficient animals.^9^ Thus, DNGR-1-dependent cross-presentation of dead cell-associated antigens can increase anticancer immunity and could be exploited clinically.

In addition to conventional chemotherapy and radiotherapy, a growing repertoire of therapies target driver mutations in cancer. An example is BRAF inhibitors, which have been used to treat BRAF-mutant melanoma.^20^ However, clinical responses are variable and often curtailed by development of drug resistance. Improving clinical response by co-opting immunity may help tackle resistance to targeted therapy and potentially improve outcomes. Indeed, NK cells are essential for effective melanoma control in mice treated with the BRAF inhibitor PLX4720^21^ and, in human paired-biopsy studies, treatment with BRAF-inhibitor increases tumor infiltration by cytotoxic CD8^+^ T cells.^22^ Here, we show that melanoma control by targeted therapy with PLX4720 requires an immunocompetent host and reveal that efficacy can be enhanced in the absence of sGSN. Thus, sGSN blocking could be a potentially useful adjunct to targeted therapy.

Collectively, these data complement and extend our previous observations that sGSN-deficient mice display enhanced responses to immune checkpoint blockade cancer therapy,^9^ and that DNGR-1 expression within tumors is associated with greater OS in several human cancers.^10^ Further highlighting the sGSN-DNGR-1 axis as a novel checkpoint in anticancer immunity, we previously reported that in a small subset of patients with stomach adenocarcinoma, high expression of *CLEC9A* and low expression of *sGSN* in tumors conferred the best survival outcomes compared with the other stratified subgroups in retrospective analysis.^9^ These findings will need to be prospectively validated to identify patients who may benefit most from inhibition of sGSN function to unleash DNGR-1-dependent cross-presentation as a component of more effective treatment regimens.

## Data Availability

Data are available on reasonable request. All data relevant to the study are included in the article or uploaded as online supplemental information.
