# Supplementary figures and images for: Loss of secreted gelsolin enhances response to anticancer therapies
Source: J Immunother Cancer. 2022 Sep 26;10(9):e005245. doi: 10.1136/jitc-2022-005245 (PMC9516286; doi:10.1136/jitc-2022-005245)

Supplementary Figure S1

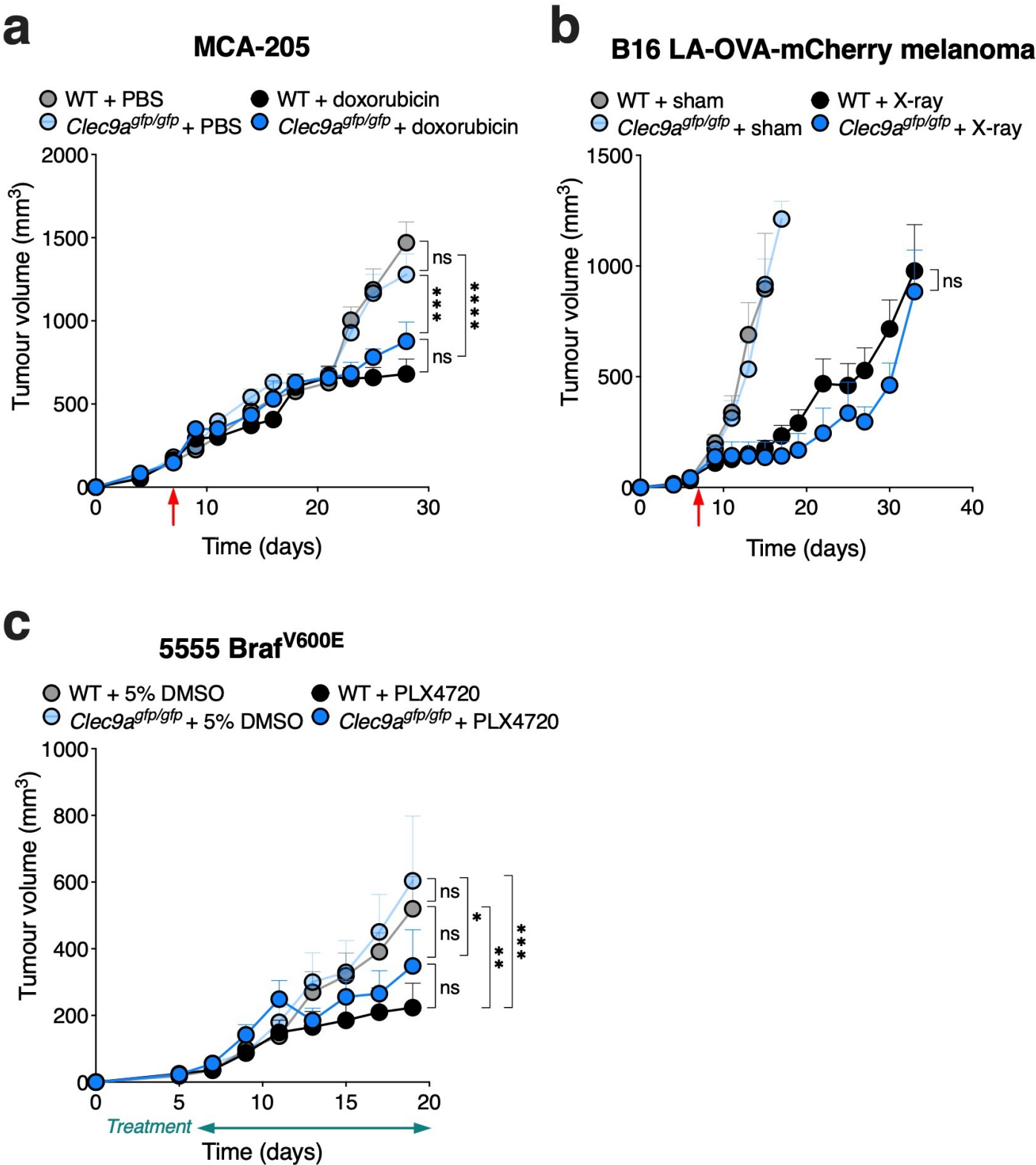

Supplement: Supplementary data [file jitc-2022-005245supp001.pdf]
