# Supplementary material for: Loss of secreted gelsolin enhances response to anticancer therapies
Source: J Immunother Cancer. 2022 Sep 26;10(9):e005245. doi: 10.1136/jitc-2022-005245 (PMC9516286; doi:10.1136/jitc-2022-005245)
Supplement: Supplementary data [file jitc-2022-005245supp002.pdf]

SUPPLEMENTARY FIGURE CAPTION

Supplementary Figure S1 | DNGR-1 is dispensable in sGSN-sufficient mice for tumour control following immunogenic chemotherapy, radiotherapy and targeted therapy. (a) Tumour growth profile in mice bearing MCA-205 fibrosarcoma receiving chemotherapy. Doxorubicin was administered to cohoused WT (*n* = 8) and *Clec9a*$^{gfp/gfp}$ (*n* = 7) mice, and compared to cohoused WT (*n* = 6) and *Clec9a*$^{gfp/gfp}$ (*n* = 7) mice receiving vehicle control. **(b)** Growth profile of B16-F10 LA-OVA-mCherry melanoma in co-housed WT (X-ray, *n* = 12; sham, *n* = 10) and *Clec9a*$^{gfp/gfp}$ (X-ray, *n* = 11; sham, *n* = 9) mice. **(c)** Growth profile of 5555 Braf^{V600E} melanoma in *Clec9a*$^{gfp/gfp}$ mice (PLX4720, *n* = 8; control, *n* = 6), compared to respective cohorts of WT mice (PLX4720, *n* = 7; control, *n* = 6) previously shown in **Figure 1h** (experiment performed at the same time). Data are plotted as tumour volume (mm^3) $\pm$ s.e.m., and mean tumour volumes were compared using two-way ANOVA with post-hoc Bonferroni correction. Where indicated, *$P \leq 0.05$, **$P < 0.01$, ***$P < 0.001$, ****$P < 0.0001$; ns, not significant. Error bars are depicted in all plots, even when not visible. Data are from one experiment.
